# High Accuracy Spline Explicit Group (SEG) Approximation for Two Dimensional Elliptic Boundary Value Problems

**DOI:** 10.1371/journal.pone.0132782

**Published:** 2015-07-16

**Authors:** Joan Goh, Norhashidah Hj. M. Ali

**Affiliations:** School of Mathematical Sciences, Universiti Sains Malaysia, Pulau Pinang, Malaysia; Georgia State University, UNITED STATES

## Abstract

Over the last few decades, cubic splines have been widely used to approximate differential equations due to their ability to produce highly accurate solutions. In this paper, the numerical solution of a two-dimensional elliptic partial differential equation is treated by a specific cubic spline approximation in the *x*-direction and finite difference in the *y*-direction. A four point explicit group (EG) iterative scheme with an acceleration tool is then applied to the obtained system. The formulation and implementation of the method for solving physical problems are presented in detail. The complexity of computational is also discussed and the comparative results are tabulated to illustrate the efficiency of the proposed method.

## Introduction

Consider the two-dimensional elliptic partial differential equation

$$\begin{array}{c} \frac{\partial^{2}u}{\partial x^{2}}+\frac{\partial^{2}u}{\partial y^{2}}=D\left(x\right)\frac{\partial u}{\partial x}+g\left(x,y\right),\;\left(x,y\right)\in \Omega \end{array}$$ (1)

which is defined in the solution domain Ω = {(*x*, *y*):0 < *x*, *y* < 1}, where functions *D*(*x*) and *g*(*x*, *y*) ∈ *C* ^2^(Ω). The corresponding Dirichlet boundary conditions are given by

$$\begin{array}{c} u(x,y)=\psi (x,y),\;(x,y)\in \partial \Omega \end{array}$$ (2)

where ∂Ω is its boundary. These types of problems arise very frequently in different areas of applied mathematics and physics such as convection-diffusion equation which describes the transport phenomena, and the Poisson’s equation which is broadly used in electrostatics, mechanical engineering and theoretical physics. Thus, solving elliptic differential equation have been of interest to many authors [1–3].

In 1968, Bickley [4] suggested the use of cubic splines for solving a linear ordinary differential. Following this, Albasiny and Hoskins [5] approximated the solutions by applying the cubic spline interpolation introduced by Ahlberg *et al.* [6], which leads to a matrix system of tri-diagonal instead of upper Hessenberg form which was obtained by Bickley [4]. The cubic spline method suggested by Bickley [4] was then examined by Fyfe [7]. Fyfe concluded that spline method is better than the usual finite difference method in terms of its accuracy and also its flexibility to get the approximation at any point in the domain. Due to its simplicity, many researchers started to work on spline methods for solving boundary value problems [8–11]. To mention a few, Bialecki *et al.* [12] formulated a new fourth order one step nodal bicubic spline collocation methods for the solution of various elliptic boundary value problems. Mohanty and Gopal [13] proposed a high accuracy cubic spline finite difference approximation of *O*(*k* ^2^ + *h* ^4^) accuracy for the solution of non-linear wave equation. Goh *et al.* [14] discussed the solution for one-dimensional heat and advection-diffusion by using a combination of finite difference approach and cubic B-spline method.

Over the last few decades, we have seen the formulation of group iterative methods for solving the two dimensional elliptic partial difference Eqs [15–18]. In 1991, a half-sweep iterative method had been introduced by Abdullah [19] via the explicit decoupled group (EDG) iterative method which was shown to be faster and computationally economical than the existing explicit group (EG) method due to Yousif and Evans [18] for solving elliptic partial differential equation. Inspired by Abdullah [19], Othman and Abdullah [20] proposed a quarter-sweep iteration through the modified explicit group (MEG) method. Following this, Ali and Ng [21] extended the idea and formulated the modified explicit decoupled group (MEDG) method for solving two-dimensional Poisson equation. The MEDG method exhibits a better convergence rather than the existing group schemes of the same family, namely EG, EDG and MEG methods.

In 1986, Yousif and Evans [18] developed the explicit group (EG) iterative method where a small group of 2, 4, 9, 16 and 25 points were constructed in the iterative processes for solving Laplace’s equation. The numerical results show that the EG method is simpler to program compared to the block (line) iterative methods and it requires less storage. However, this method was solely formulated using the usual standard finite difference discretization which restricts the solutions at only certain points of the solution domain. This, thus, motivate us to adopt the idea in using splines in the formulation of the group methods.

In this paper, a new method, namely spline explicit group (SEG) iterative method, which incorporates cubic spline with group iterative scheme, is developed for solving the elliptic problems. Using a cubic spline approximation in the *x*-direction and central difference in the *y*-direction, we obtain a new three level implicit nine-point compact finite difference formulation. Then, a four point explicit group iterative scheme is applied to the obtained system. The performance of the method will be investigated via two benchmark problems, that is the convection-diffusion equation and Poisson’s equation.

## The Cubic Spline Approximation and Numerical Scheme

Here, the solution domain Ω = [0, 1] × [0, 1] is divided by *h* > 0 in *x*-direction and *k* > 0 in *y*-direction. Therefore, the grid points (*x*_*l*_, *y*_*m*_) are represented as *x*_*l*_ = *lh* and *y*_*m*_ = *mk*, *l* = 0, 1, …, *N*_*x*_, *m* = 0, 1, …, *N*_*y*_, where *N*_*x*_ and *N*_*y*_ are positive integers. Let *U*_*l*, *m*_ be the approximation solution of *u*_*l*, *m*_ at the grid point (*x*_*l*_, *y*_*m*_).

Suppose that *S*_*m*_(*x*) is the *m*-th mesh row cubic spline polynomial which interpolates the value *U*_*l*, *m*_ at (*x*_*l*_, *y*_*m*_), is given by [6]

$$\begin{array}{c} \begin{array}{cc} S_{m}\left(x\right)= & \frac{{\left(x_{l}-x\right)}^{3}}{6h}M_{l-1,m}+\frac{{\left(x-x_{l-1}\right)}^{3}}{6h}M_{l,m}+(\frac{x_{l}-x}{h})(U_{l-1,m}-\frac{h^{2}}{6}M_{l-1,m})\\ & +(\frac{x-x_{l-1}}{h})(U_{l,m}-\frac{h^{2}}{6}M_{l,m}) \end{array} \end{array}$$ (3)

for *x*_*l*−1_ ≤ *x* ≤ *x*_*l*_, where *l* = 1, 2, …, *N*_*x*_ and *m* = 0, 1, 2, …, *N*_*y*_. For each *m*-th mesh row, the cubic spline *S*_*m*_(*x*) satisfies the following properties

1. *S*_*m*_(*x*) coincides with a polynomial of degree three on each [*x*_*l*−1_, *x*_*l*_], *l* = 1, 2, …, *N*_*x*_, *m* = 0, 1, 2, …, *N*_*y*_
2. *S*_*m*_(*x*) ∈ *C* ^2^[0, 1], and
3. *S*_*m*_(*x*_*l*_) = *U*_*l*, *m*_, *l* = 0, 1, 2, …, *N*_*x*_, *m* = 0, 1, 2, …, *N*_*y*_

The derivatives of cubic spline *S*_*m*_(*x*) can be obtained as below

$$\begin{array}{c} S_{m}^{\prime }\left(x\right)=\frac{-{\left(x_{l}-x\right)}^{2}}{2h}M_{l-1,m}+\frac{{\left(x-x_{l-1}\right)}^{2}}{2h}M_{l,m}+\frac{U_{l,m}-U_{l-1,m}}{h}-\frac{h}{6}[M_{l,m}-M_{l-1,m}] \end{array}$$ (4)

$$\begin{array}{c} S_{m}^{\prime \prime }\left(x\right)=\frac{\left(x_{l}-x\right)}{h}M_{l-1,m}+\frac{\left(x-x_{l-1}\right)}{h}M_{l,m} \end{array}$$ (5)

And, from Eq (1), it gives

$$\begin{array}{c} M_{l,m}=S_{m}^{\prime \prime }\left(x_{l}\right)=U_{xxl,m}=-U_{yyl,m}+D_{l}U_{xl,m}+g_{l,m} \end{array}$$ (6)

When *x* = *x*_*l*_, Eq (4) becomes

$$\begin{array}{c} S_{m}^{\prime }\left(x_{l}\right)=U_{xl,m}=\frac{U_{l,m}-U_{l-1,m}}{h}+\frac{h}{6}[M_{l-1,m}+2M_{l,m}] \end{array}$$ (7)

Similarly, for *x* ∈ [*x*_*l*_, *x*_*l*+1_], it gives

$$\begin{array}{c} S_{m}^{\prime }\left(x_{l}\right)=U_{xl,m}=\frac{U_{l+1,m}-U_{l,m}}{h}-\frac{h}{6}[M_{l+1,m}+2M_{l,m}] \end{array}$$ (8)

Combining both Eqs (7) and (8), the following approximation can be obtained

$$\begin{array}{c} S_{m}^{\prime }\left(x_{l}\right)=U_{xl,m}=\frac{U_{l+1,m}-U_{l-1,m}}{2h}-\frac{h}{12}[M_{l+1,m}-M_{l-1,m}] \end{array}$$ (9)

Further, we have

$$S_{m}^{\prime }\left(x_{l+1}\right)=U_{xl+1,m}=\frac{U_{l+1,m}-U_{l,m}}{h}+\frac{h}{6}\left[M_{l,m}+2M_{l+1,m}\right]$$ (10)

$$S_{m}^{\prime }\left(x_{l-1}\right)=U_{xl-1,m}=\frac{U_{l,m}-U_{l-1,m}}{h}-\frac{h}{6}\left[M_{l,m}+2M_{l-1,m}\right]$$ (11)

By using the continuity of first derivative at (*x*_*l*_, *y*_*m*_), which is, $S_{{}^{{}_{m}}}^{\prime }(x_{l}^{+})=S_{{}^{{}_{m}}}^{\prime }(x_{l}^{-})$, the following relation can be obtained

$$\begin{array}{c} U_{l+1,m}-2U_{l,m}+U_{l-1,m}=\frac{h^{2}}{6}(M_{l+1,m}+4M_{l,m}+M_{l-1,m}) \end{array}$$ (12)

The following approximations are considered

$$\begin{array}{l} {\bar{U}}_{yyl,m}=\left(U_{l,m+1}-2U_{l,m}+U_{l,m-1}\right)/k^{2} \end{array}$$ (13a)

$$\begin{array}{l} {\bar{U}}_{yyl+1,m}=\left(U_{l+1,m+1}-2U_{l+1,m}+U_{l+1,m-1}\right)/k^{2} \end{array}$$ (13b)

$$\begin{array}{l} {\bar{U}}_{yyl-1,m}=\left(U_{l-1,m+1}-2U_{l-1,m}+U_{l-1,m-1}\right)/k^{2} \end{array}$$ (13c)

$$\begin{array}{l} {\bar{U}}_{xl,m}=\left(U_{l+1,m}-U_{l-1,m}\right)/\left(2h\right) \end{array}$$ (14a)

$$\begin{array}{l} {\bar{U}}_{xl+1,m}=\left(3U_{l+1,m}-4U_{l,m}+U_{l-1,m}\right)/\left(2h\right) \end{array}$$ (14b)

$$\begin{array}{l} {\bar{U}}_{xl-1,m}=\left(-3U_{l-1,m}+4U_{l,m}-U_{l+1,m}\right)/\left(2h\right) \end{array}$$ (14c)

Eqs (14b) and (14c) are obtained from the second-order one-sided finite difference scheme. For the derivatives of *S*_*m*_(*x*), we consider

$$\begin{array}{l} {\bar{M}}_{l,m}=-{\bar{U}}_{yyl,m}+D_{l}{\bar{U}}_{xl,m}+g_{l,m} \end{array}$$ (15a)

$$\begin{array}{l} {\bar{M}}_{l+1,m}=-{\bar{U}}_{yyl+1,m}+D_{l+1}{\bar{U}}_{xl+1,m}+g_{l+1,m} \end{array}$$ (15b)

$$\begin{array}{l} {\bar{M}}_{l-1,m}=-{\bar{U}}_{yyl-1,m}+D_{l-1}{\bar{U}}_{xl-1,m}+g_{l-1,m} \end{array}$$ (15c)

$$\begin{array}{l} {\bar{\bar{U}}}_{xl+1,m}=\frac{U_{l+1,m}-U_{l,m}}{h}+\frac{h}{6}\left[{\bar{M}}_{l,m}+2{\bar{M}}_{l+1,m}\right] \end{array}$$ (15d)

$$\begin{array}{l} {\bar{\bar{U}}}_{xl-1,m}=\frac{U_{l,m}-U_{l-1,m}}{h}-\frac{h}{6}\left[{\bar{M}}_{l,m}+2{\bar{M}}_{l-1,m}\right] \end{array}$$ (15e)

$$\begin{array}{l} {\hat{U}}_{xl,m}=\frac{U_{l+1,m}-U_{l-1,m}}{2h}-\frac{h}{12}\left[{\bar{M}}_{l+1,m}-{\bar{M}}_{l-1,m}\right] \end{array}$$ (15f)

By using Taylor series expansion about the grid point (*x*_*l*_, *y*_*m*_), Eq (1) can be written as

$$\begin{array}{c} \begin{array}{cc} & \left(U_{l+1,m}-2U_{l,m}+U_{l-1,m})+\frac{h^{2}}{12}[{\bar{U}}_{yyl+1,m}+{\bar{U}}_{yyl-1,m}+10{\bar{U}}_{yyl,m}\right]\\ = & \frac{h^{2}}{12}[D_{l+1}{\bar{\bar{U}}}_{xl+1,m}+D_{l-1}{\bar{\bar{U}}}_{xl-1,m}+10D_{l}{\hat{U}}_{xl,m}]+\frac{h^{2}}{12}[g_{l+1,m}+g_{l-1,m}+10g_{l,m}]+T_{l,m} \end{array} \end{array}$$ (16)

where *T*_*l*, *m*_ is the local truncation error. Substituting the above approximations (13)–(15) into (16), it results

$$\begin{array}{c} \begin{array}{cc} & \{-2+12\lambda^{2}-\lambda^{2}h\left(D_{l+1}+5D_{l}\right)+\frac{\lambda^{2}h^{2}}{12}[\left(5D_{l}-2D_{l-1}\right)D_{l-1}-3\left(2D_{l+1}-5D_{l}\right)D_{l+1}\\ & -\left(D_{l+1}-D_{l-1}\right)D_{l}]-\frac{h}{3}\left(2D_{l+1}-5D_{l}\right)\}U_{l+1,m}\\ & +\{-20-24\lambda^{2}-\lambda^{2}h\left(D_{l-1}-D_{l+1}\right)+\frac{\lambda^{2}h^{2}}{3}[\left(2D_{l+1}-5D_{l}\right)D_{l+1}-\left(5D_{l}-2D_{l-1}\right)D_{l-1}]\\ & -\frac{h}{3}\left(D_{l+1}-D_{l-1}\right)\}U_{l,m}\\ & +\{-2+12\lambda^{2}+\lambda^{2}h\left(5D_{l}+D_{l-1}\right)+\frac{\lambda^{2}h^{2}}{12}[3\left(5D_{l}-2D_{l-1}\right)D_{l-1}-\left(2D_{l+1}-5D_{l}\right)D_{l+1}\\ & +\left(D_{l+1}-D_{l-1}\right)D_{l}]-\frac{h}{3}\left(5D_{l}-2D_{l-1}\right)\}U_{l-1,m}\\ & +[1+\frac{h}{6}\left(2D_{l+1}-5D_{l}\right)](U_{l+1,m+1}+U_{l+1,m-1})\\ & +[1+\frac{h}{6}\left(5D_{l}-2D_{l-1}\right)]\left(U_{l-1,m+1}+U_{l-1,m-1}\right)\\ & +[10+\frac{h}{6}\left(D_{l+1}-D_{l-1}\right)]\left(U_{l,m+1}+U_{l,m-1}\right)\\ = & k^{2}\{[1+\frac{h}{6}\left(2D_{l+1}-5D_{l}\right)]g_{l+1,m}+[1+\frac{h}{6}\left(5D_{l}-2D_{l-1}\right)]g_{l-1,m}\\ & +[10+\frac{h}{6}\left(D_{l+1}-D_{l-1}\right)]g_{l,m}\}+T_{l,m} \end{array} \end{array}$$ (17)

where *λ* is the mesh ratio, denoted by *λ* = (*k*/*h*). If the singular terms like $\frac{1}{x}$ appear in the functions *D*(*x*) and/or *g*(*x*, *y*), which is unable to evaluate at *x* = 0. The following approximations are considered

$$\begin{array}{cc} & D_{l\pm 1}=D_{00}\pm hD_{10}+\frac{h^{2}}{2}D_{20}\pm O\left(h^{3}\right) \end{array}$$ (18a)

$$\begin{array}{ll} & g_{l\pm 1,m}=g_{00}\pm hg_{10}+\frac{h^{2}}{2}g_{20}\pm O\left(h^{3}\right) \end{array}$$ (18b)

where

$$\begin{array}{cc} & W_{ab}=\frac{\partial^{a+b}W\left(x_{l},y_{m}\right)}{\partial x^{a}\partial y^{b}},\;W\;=\;D\;\text{and}\;g \end{array}$$

Thus, neglecting the higher order terms and local truncation error, Eq (17) can be written as

$$\begin{array}{c} \begin{array}{cc} & \{-2+12\lambda^{2}-\frac{1}{2}\lambda^{2}h\left(12D_{00}+h^{2}D_{20}\right)-\lambda^{2}h^{2}\left(D_{10}-D_{00}D_{00}\right)+hD_{00}\}U_{l+1,m}\\ + & \{-20-24\lambda^{2}+2\lambda^{2}h^{2}\left(D_{10}-D_{00}D_{00}\right)-\frac{2}{3}h^{2}D_{10}\}U_{l,m}\\ + & \{-2+12\lambda^{2}+\frac{1}{2}\lambda^{2}h\left(12D_{00}+h^{2}D_{20}\right)+\lambda^{2}h^{2}\left(D_{00}D_{00}-D_{10}\right)-hD_{00}\}U_{l-1,m}\\ + & (1-\frac{h}{2}D_{00})\left(U_{l+1,m+1}+U_{l+1,m-1}\right)+(1+\frac{h}{2}D_{00})\left(U_{l-1,m+1}+U_{l-1,m-1}\right)\\ + & (10+\frac{h^{2}}{3}D_{10})\left(U_{l,m+1}+U_{l,m-1}\right)\\ = & k^{2}[12g_{00}+h^{2}\left(g_{20}+D_{10}g_{00}-D_{00}g_{10}\right)]=G_{l,m} \end{array} \end{array}$$ (19)

This modified equation retains its order of accuracy everywhere throughout the solution region, moreover in the vicinity of the singularity. Note that, this proposed scheme (19) is of *O*(*k* ^2^ + *k* ^2^ *h* ^2^ + *h* ^4^) and applicable to both singular and non-singular elliptic equations of the form (1).

## Spline Explicit Group Method

**Fig 1:**
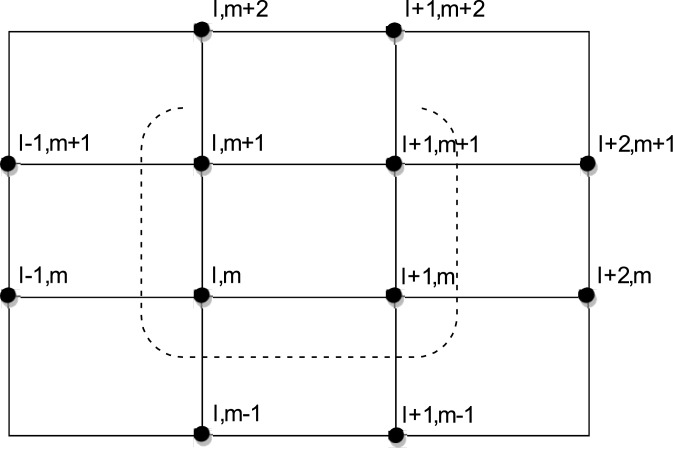
Computational Molecule of Eq (20).

We apply Eq (19) to any group of four points on the solution domain (as shown in Fig 1). Then, a (4 × 4) system as below, can be obtained

$$\begin{array}{c} \left[\begin{array}{cccc} a_{1} & a_{2} & a_{3} & a_{4}\\ a_{5} & a_{1} & a_{4} & a_{6}\\ a_{6} & a_{4} & a_{1} & a_{5}\\ a_{4} & a_{3} & a_{2} & a_{1} \end{array}][\begin{array}{c} U_{l,m}\\ U_{l+1,m}\\ U_{l+1,m+1}\\ U_{l,m+1} \end{array}]=[\begin{array}{c} rhs_{l,m}\\ rhs_{l+1,m}\\ rhs_{l+1,m+1}\\ rhs_{l,m+1} \end{array}\right] \end{array}$$ (20)

where

$$\begin{array}{cc} & a_{1}=-20-24\lambda^{2}+2\lambda^{2}h^{2}\left(D_{10}-D_{00}D_{00}\right)-\frac{2}{3}h^{2}D_{10}\\ & a_{2}=-2+12\lambda^{2}-\frac{1}{2}\lambda^{2}h\left(12D_{00}+h^{2}D_{20}\right)-\lambda^{2}h^{2}\left(D_{10}-D_{00}D_{00}\right)+hD_{00}\\ & a_{3}=1-\frac{h}{2}D_{00}\\ & a_{4}=10+\frac{h^{2}}{3}D_{10}\\ & a_{5}=-2+12\lambda^{2}+\frac{1}{2}\lambda^{2}h\left(12D_{00}+h^{2}D_{20}\right)+\lambda^{2}h^{2}\left(D_{00}D_{00}-D_{10}\right)-hD_{00}\\ & a_{6}=1+\frac{h}{2}D_{00} \end{array}$$

and

$$\begin{array}{cc} & rhs_{l,m}=-a_{5}U_{l-1,m}-a_{3}U_{l+1,m-1}-a_{6}\left(U_{l-1,m+1}+U_{l-1,m-1}\right)-a_{4}U_{l,m-1}+G_{l,m}\\ & rhs_{l+1,m}=-a_{2}U_{l+2,m}-a_{3}\left(U_{l+2,m+1}+U_{l+2,m-1}\right)-a_{6}U_{l,m-1}-a_{4}U_{l+1,m-1}+G_{l+1,m}\\ & rhs_{l+1,m+1}=-a_{2}U_{l+2,m+1}-a_{3}\left(U_{l+2,m+2}+U_{l+2,m}\right)-a_{6}U_{l,m+2}-a_{4}U_{l+1,m+2}+G_{l+1,m+1}\\ & rhs_{l,m+1}=-a_{5}U_{l-1,m+1}-a_{3}U_{l+1,m+2}-a_{6}\left(U_{l-1,m+2}+U_{l-1,m}\right)-a_{4}U_{l,m+2}+G_{l,m+1} \end{array}$$

Eq (20) can be inverted and written in explicit forms

$$\begin{array}{c} \left[\begin{array}{c} U_{l,m}\\ U_{l+1,m}\\ U_{l+1,m+1}\\ U_{l,m+1} \end{array}]=\frac{1}{denom}[\begin{array}{cccc} b_{1} & b_{2} & b_{3} & b_{4}\\ b_{5} & b_{1} & b_{4} & b_{6}\\ b_{6} & b_{4} & b_{1} & b_{5}\\ b_{4} & b_{3} & b_{2} & b_{1} \end{array}][\begin{array}{c} rhs_{l,m}\\ rhs_{l+1,m}\\ rhs_{l+1,m+1}\\ rhs_{l,m+1} \end{array}\right] \end{array}$$ (21)

where

$$\begin{array}{cc} denom= & a_{1}^{4}-2a_{1}^{2}a_{4}^{2}+a_{4}^{4}-2a_{1}^{2}a_{2}a_{5}+4a_{1}a_{3}a_{4}a_{5}-2a_{2}a_{4}^{2}a_{5}+a_{2}^{2}a_{5}^{2}-a_{3}^{2}a_{5}^{2}-2a_{1}^{2}a_{3}a_{6}\\ & +4a_{1}a_{2}a_{4}a_{6}-2a_{3}a_{4}^{2}a_{6}-a_{2}^{2}a_{6}^{2}+a_{3}^{2}a_{6}^{2} \end{array}$$

and

$$\begin{array}{cc} & b_{1}=a_{1}^{3}-a_{1}a_{4}^{2}-a_{1}a_{2}a_{5}+a_{3}a_{4}a_{5}-a_{1}a_{3}a_{6}+a_{2}a_{4}a_{6}\\ & b_{2}=-a_{1}^{2}a_{2}+2a_{1}a_{3}a_{4}-a_{2}a_{4}^{2}+a_{2}^{2}a_{5}-a_{3}^{2}a_{5}\\ & b_{3}=-a_{1}^{2}a_{3}+2a_{1}a_{2}a_{4}-a_{3}a_{4}^{2}-a_{2}^{2}a_{6}+a_{3}^{2}a_{6}\\ & b_{4}=-a_{1}^{2}a_{4}+a_{4}^{3}+a_{1}a_{3}a_{5}-a_{2}a_{4}a_{5}+a_{1}a_{2}a_{6}-a_{3}a_{4}a_{6}\\ & b_{5}=-a_{1}^{2}a_{5}-a_{4}^{2}a_{5}+a_{2}a_{5}^{2}+2a_{1}a_{4}a_{6}-a_{2}a_{6}^{2}\\ & b_{6}=2a_{1}a_{4}a_{5}-a_{3}a_{5}^{2}-a_{1}^{2}a_{6}-a_{4}^{2}a_{6}+a_{3}a_{6}^{2} \end{array}$$

The Gauss-Seidel technique is employed to accelerate the convergence process. Iterations are generated in groups of four points over the entire spatial domain until the convergence test is satisfied. Once the approximations *U*_*l*,*m*_ had been calculated, the value of *M*_*l*,*m*_ can be easily obtained by solving the system generated by (12). Then, the piecewise polynomial of the function can be obtained from Eq (3). Finally, the approximate solution at any point at *m*-th mesh row can be easily calculated.

**Fig 2:**
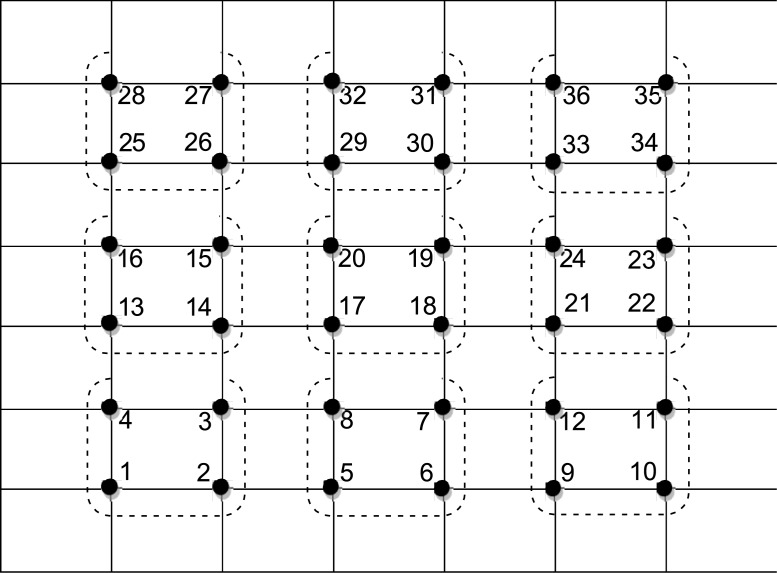
Points ordering for SEG method.

Applying Eq (21) to each of the group in natural row ordering (Fig 2) will lead to a linear system

AU=b

where the matrix of coefficient *A* is given by

$$\begin{array}{c} A=[\begin{array}{ccc} D & V & \\ L & D & V\\ & L & D \end{array}] \end{array}$$ (22)

with

$$D=[\begin{array}{ccc} R_{0} & R_{2} & \\ R_{5} & R_{0} & R_{2}\\ & R_{5} & R_{0} \end{array}],\;V=[\begin{array}{ccc} R_{4} & R_{3} & \\ R_{6} & R_{4} & R_{3}\\ & R_{6} & R_{4} \end{array}],\;L=[\begin{array}{ccc} R_{4}^{\prime } & R_{3}^{\prime } & \\ R_{6}^{\prime } & R_{4}^{\prime } & R_{3}^{\prime }\\ & R_{6}^{\prime } & R_{4}^{\prime } \end{array}]$$

The submatrices are given by

$$R_{0}=[\begin{array}{cccc} a_{1} & a_{2} & a_{3} & a_{4}\\ a_{5} & a_{1} & a_{4} & a_{6}\\ a_{6} & a_{4} & a_{1} & a_{5}\\ a_{4} & a_{3} & a_{2} & a_{1} \end{array}],\;R_{2}=[\begin{array}{cccc} 0 & 0 & 0 & 0\\ a_{2} & 0 & 0 & a_{3}\\ a_{3} & 0 & 0 & a_{2}\\ 0 & 0 & 0 & 0 \end{array}],\;R_{3}=[\begin{array}{cccc} 0 & 0 & 0 & 0\\ 0 & 0 & 0 & 0\\ a_{3} & 0 & 0 & 0\\ 0 & 0 & 0 & 0 \end{array}],$$

$$R_{4}=[\begin{array}{cccc} 0 & 0 & 0 & 0\\ 0 & 0 & 0 & 0\\ a_{6} & a_{4} & 0 & 0\\ a_{4} & a_{3} & 0 & 0 \end{array}],\;R_{5}=[\begin{array}{cccc} 0 & a_{5} & a_{6} & 0\\ 0 & 0 & 0 & 0\\ 0 & 0 & 0 & 0\\ 0 & a_{6} & a_{5} & 0 \end{array}],\;R_{6}=[\begin{array}{cccc} 0 & 0 & 0 & 0\\ 0 & 0 & 0 & 0\\ 0 & 0 & 0 & 0\\ 0 & a_{6} & 0 & 0 \end{array}],$$

$$R_{3}^{\prime }=[\begin{array}{cccc} 0 & 0 & 0 & 0\\ 0 & 0 & 0 & a_{3}\\ 0 & 0 & 0 & 0\\ 0 & 0 & 0 & 0 \end{array}],\;R_{4}^{\prime }=[\begin{array}{cccc} 0 & 0 & a_{3} & a_{4}\\ 0 & 0 & a_{4} & a_{6}\\ 0 & 0 & 0 & 0\\ 0 & 0 & 0 & 0 \end{array}],\;R_{6}^{\prime }=[\begin{array}{cccc} 0 & 0 & a_{6} & 0\\ 0 & 0 & 0 & 0\\ 0 & 0 & 0 & 0\\ 0 & 0 & 0 & 0 \end{array}]$$

In order to derive the explicit formulae, the matrix *A* is transformed into *A* ^*E*^ and vector *b* is modified into *b* ^*E*^, where,

$$\begin{array}{cc} & A^{E}=diag\left\{R_{0}^{-1}\right\}A\\ & b^{E}=diag\left\{R_{0}^{-1}\right\}b \end{array}$$

The block structure of *A* ^*E*^ is the same as matrix *A* with the nonzero block *R*_0_ replaced by identity matrices, *I* and the blocks *R*_*i*_ and $R_{j}^{\prime }$, replaced by $R_{0}^{-1}R_{i}$, *i* = 0, 2, 3, 4, 5, 6 and $R_{0}^{-1}R_{{}^{{}_{j}}}^{\prime }$, *j* = 3,4,6 respectively. Since the coefficient matrix (22) is block tridiagonal with non-vanishing diagonal element, it is *π*-consistently ordered and has property-*A* ^(*π*)^ [22]. Thus, the theory of block S.O.R. is also applicable to the SEG iterative method and therefore, is convergent.

## Computational Complexity Analysis

**Fig 3:**
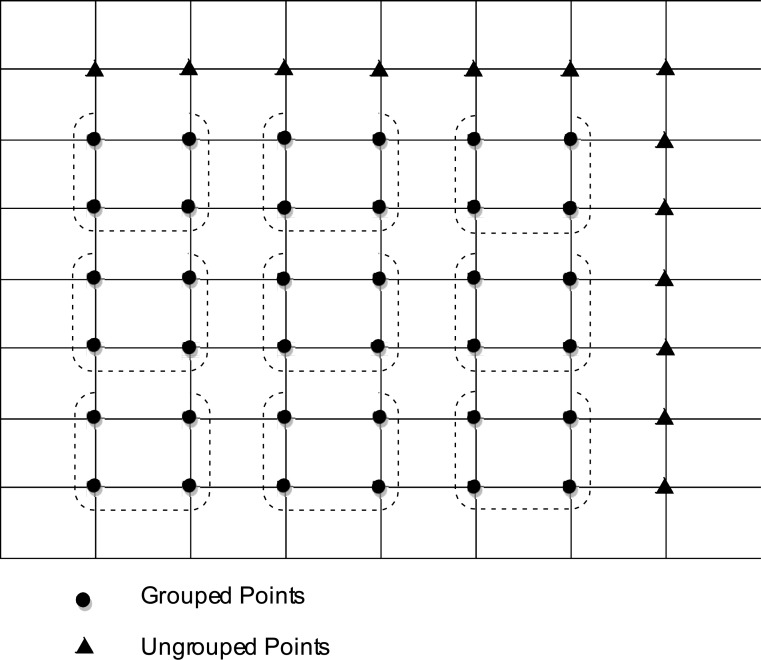
Types of points in SEG for *N*_*x*_ = *N*_*y*_ = 8.

In order to show the efficiency of the proposed method, computational complexity of the SEG iterative method is examined. Assume that the solution domain is discretized into even intervals, *N*_*x*_ and *N*_*y*_ in *x*- and *y*-directions, respectively. Therefore, we have (*n*_*x*_ − 1)(*n*_*y*_ − 1) grouped points and (*n*_*x*_ + *n*_*y*_ − 1) ungrouped points, where *n*_*x*_ = *N*_*x*_ − 1 and *n*_*y*_ = *N*_*y*_ − 1. This can be shown as in Fig 3.

The estimation on this computational complexity is based on the arithmetic operations performed at each iteration for the additions/substractions (Add/Sub) and multiplications/divisions (Mul/Div) operations. Therefore, the number of operations required for SEG is given as in Table 1. The total number of arithmetic operations can be obtained by multiplying the number of arithmetic operations for each iteration with the number of iterations.

**Table 1:** The number of arithmetic operations per iteration for SEG iterative method.

	Internal Points	Add/Sub	Mul/Div
Grouped Points	(*n*_*x*_ − 1)(*n*_*y*_ − 1)	8(*n*_*x*_ − 1)(*n*_*y*_ − 1)	8(*n*_*x*_ − 1)(*n*_*y*_ − 1)
Ungrouped Points	(*n*_*x*_ + *n*_*y*_ − 1)	8(*n*_*x*_ + *n*_*y*_ − 1)	6(*n*_*x*_ + *n*_*y*_ − 1)
Total	*n*_*x*_ *n*_*y*_	8*n*_*x*_ *n*_*y*_	8*n*_*x*_ *n*_*y*_ − 2(*n*_*x*_ + *n*_*y*_ − 1)

## Numerical Results

In this section, two benchmark test problems, whose exact solutions are known are solved by the proposed combination of cubic spline and explicit group iterative method. The results are then compared with those obtained by the

- Combination of cubic spline with block Gauss-Seidel iterative method (SBGS)
- Combination of central difference scheme with explicit group iterative method (CDEG)

where the CDEG scheme can be derived by substituting the partial derivative in Eq (1) by the central difference approximation. In all cases, we assume that *u* ^(0)^ = 0 as the initial guess and the iterations were stopped when the estimated error was below tolerance, that is when ∣*u* ^(*s*+1)^ − *u* ^(*s*)^∣ ≤ 10^−12^ was achieved.

### Example 1

Consider the convection-diffusion equation

$$\begin{array}{cc} & \frac{\partial^{2}u}{\partial x^{2}}+\frac{\partial^{2}u}{\partial y^{2}}=\beta \frac{\partial u}{\partial x},\;0<x,y<1 \end{array}$$

The exact solution for the problem is given by

$$u\left(x,y\right)=e^{\frac{\beta x}{2}}\frac{\sin\pi y}{\sinh\sigma }[2e^{\frac{-\beta }{2}}\sinh\sigma x+\sinh\sigma \left(1-x\right)]$$

where $\sigma^{2}=\pi^{2}+\frac{\beta^{2}}{4}$ and *β* > 0. The boundary conditions can be obtained from the exact solution. The (4 × 4) matrix system can be obtained by substituting *D*_00_ = *β*, *D*_10_ = *D*_20_ = 0 and *G*_*l*, *m*_ = *G*_*l*+1, *m*_ = *G*_*l*+1, *m*+1_ = *G*_*l*, *m*+1_ = 0 in Eq (20). The maximum absolute errors are tabulated in Table 2 and the number of arithmetic operations are shown in Table 3.

**Table 2:** Computational errors for proposed method, SEG compared with CDEG [18] and SBGS [23] by using *β* = 10 and *k*/*h* ^2^ = 64.

*h*	**CDEG**		**SBGS**		**SEG**	
	Maximum Absolute Errors	Time (Seconds)	Maximum Absolute Errors	Time (Seconds)	Maximum Absolute Errors	Time (Seconds)
1/16	1.27722E-02	0.03	1.63610E-02	0.02	1.63610E-02	0.01
1/32	3.56699E-03	0.21	1.02672E-03	0.23	1.02672E-03	0.22
1/64	1.03777E-03	18.40	6.42077E-05	25.16	6.42077E-05	18.40

**Table 3:** Total arithmetic operations needed to generate the above results for CDEG [18], SBGS [23] and the proposed method, SEG (*β* = 10, *k*/*h* ^2^ = 64).

*h*	**CDEG**		**SBGS**		**SEG**	
	Number of Iterations	Total Arithmetic Operations	Number of Iterations	Total Arithmetic Operations	Number of Iterations	Total Arithmetic Operations
1/16	119	47,481	16	26,832	123	78,843
1/32	376	1,697,640	163	5,239,635	373	2,568,105
1/64	2245	88,262,175	2261	1,194,099,669	2159	127,996,315

### Example 2

Given the following Poisson’s equation in polar cylindrical coordinates in *r* − *z* plane.

$$\begin{array}{cc} & \frac{\partial^{2}u}{\partial r^{2}}+\frac{\partial^{2}u}{\partial z^{2}}+\frac{1}{r}\frac{\partial u}{\partial r}=\cosh z(5r\cosh r+2(2+r^{2})\sinh r),\;0<r,z<1 \end{array}$$

The exact solution is *u*(*r*, *z*) = *r* ^2^ sinh *r* cosh *z*. The solutions can be approximated by replacing the variables (*x*, *y*) by (*r*, *z*) and substituting $D(r)=-\frac{1}{r}$ and *g*(*r*, *z*) = cosh *z*(5*r* cosh *r* + 2(2 + *r* ^2^) sinh *r*) into scheme (20). The corresponding errors and the number of arithmetic operations are tabulated in Tables 4 and 5, respectively.

**Table 4:** Maximum absolute errors of proposed method, SEG compared with CDEG [18] and SBGS [23] compared to the exact solution (*k*/*h* = 0.8).

*h*	**CDEG**		**SBGS**		**SEG**	
	Maximum Absolute Errors	Time (Seconds)	Maximum Absolute Errors	Time (Seconds)	Maximum Absolute Errors	Time (Seconds)
1/16	1.73383E-03	0.52	6.90966E-05	0.92	6.37611E-05	0.23
1/32	4.59371E-04	3.61	1.05767E-05	14.34	9.51966E-06	3.61
1/64	1.20177E-04	92.87	1.55798E-06	271.72	1.36015E-06	91.43
1/128	3.12015E-05	3329.25	2.23341E-07	7330.74	1.87608E-07	3302.78

**Table 5:** Total arithmetic operations needed to generate the above results for CDEG [18], SBGS [23] and the proposed method, SEG for *k*/*h* = 0.8.

*h*	**CDEG**		**SBGS**		**SEG**	
	Number of Iterations	Total Arithmetic Operations	Number of Iterations	Total Arithmetic Operations	Number of Iterations	Total Arithmetic Operations
1/16	456	1,384,416	550	5,998,300	439	1,972,866
1/32	1724	22,570,608	2094	177,541,884	1658	31,843,548
1/64	6524	354,409,776	7930	5,291,165,620	6263	496,969,050
1/128	24579	5,438,546,172	29849	157,937,088,498	23561	7,598,846,598

## Conclusions

In this paper, a new method namely, the SEG iterative method was formulated for solving the elliptic boundary value problems. The presented results show that the proposed method is capable of approximating the solution very well in terms of accuracy and execution time. It can be seen that the computation cost is reduced substantially compared to those obtained by the cubic spline block Gauss-Seidel iterative method [23], especially when the grid size increases. Furthermore, in terms of accuracy, the proposed method is superior to the original central difference explicit group iterative method [18]. In conclusion, the proposed method is a viable alternative approximation tool for solving the elliptic partial differential equations.

## References

[1] Aarão J, Bradshaw-Hajek BH, Miklavcic SJ, Ward DA. The extended domain eigenfunction method for solving elliptic boundary value problems with annular domains. Journal of Physics A: Mathematical and Theoretical. 2010;43:185202 10.1088/1751-8113/43/18/185202

[2] Malavi-Arabshahi SM, Dehghan M. Preconditioned techniques for solving large sparse linear systems arising from the discretization of the elliptic partial differential equations. Applied Mathematics and Computation. 2007;188(2):1371–1388. 10.1016/j.amc.2006.11.028

[3] Wang YM, Guo BY. Fourth-order compact finite difference method for fourth-order nonlinear elliptic boundary value problems. Journal of Computational and Applied Mathematics. 2008;221(1):76–97. 10.1016/j.cam.2007.10.007

[4] Bickley WG. Piecewise cubic interpolation and two-point boundary problems. The Computer Journal. 1968;11(2):206–208. 10.1093/comjnl/11.2.206

[5] Albasiny EL, Hoskins WD. Cubic spline solutions to two-point boundary value problems. The Computer Journal. 1969;12(2):151–153. 10.1093/comjnl/12.2.151

[6] Ahlberg JH, Nilson EN, Walsh JL. The theory of splines and their applications. Academic Press; 1967.

[7] Fyfe DJ. The use of cubic splines in the solution of two-point boundary value problems. The Computer Journal. 1969;12(2):188–192. 10.1093/comjnl/12.2.188

[8] Al-Said EA, Noor MA, Rassias TM. Cubic splines method for solving fourth-order obstacle problems. Applied Mathematics and Computation. 2006;174(1):180–187. 10.1016/j.amc.2005.03.022

[9] Goh J, Majid AA, Ismail AIM. Numerical method using cubic B-spline for the heat and wave equation. Computers & Mathematics with Applications. 2011;62(12):4492–4498. 10.1016/j.camwa.2011.10.028

[10] Goh J, Majid AA, Ismail AIM. A quartic B-spline for second-order singular boundary value problems. Computers & Mathematics with Applications. 2012;64(2):115–120. 10.1016/j.camwa.2012.01.022

[11] Ding HF, Zhang YX, Cao JX, Tian JH. A class of difference scheme for solving telegraph equation by new non-polynomial spline methods. Applied Mathematics and Computation. 2012;218(9):4671–4683. 10.1016/j.amc.2011.10.078

[12] Bialecki B, Fairweather G, Karageorghis A. Optimal superconvergent one step nodal cubic spline collocation methods. SIAM Journal on Scientific Computing. 2005;27(2):575–598. 10.1137/040609793

[13] Mohanty RK, Gopal V. High accuracy cubic spline finite difference approximation for the solution of one-space dimensional non-linear wave equations. Applied Mathematics and Computation. 2011;218(8):4234–4244. 10.1016/j.amc.2011.09.054

[14] Goh J, Majid AA, Ismail AIM. Cubic B-Spline collocation method for one-dimensional heat and advection-diffusion equations. Journal of Applied Mathematics. 2012;2012 10.1155/2012/458701

[15] Ali NHM, Foo KP. Modified Explicit Group AOR Methods in the solution of elliptic equations. Applied Mathematical Sciences. 2012;6:2465–2480.

[16] Evans DJ, Abdullah AR. The group explicit method for the solution of Burger’s equation. Computing. 1984;32(3):239–253. 10.1007/BF02243575

[17] Saeed AM, Ali NHM. Preconditioned modified explicit decoupled group method in the solution of elliptic PDEs Applied Mathematical Sciences. 2010;p. 1165–1181.

[18] Yousif WS, Evans DJ. Explicit group over-relaxation methods for solving elliptic partial differential equations. Mathematics and Computers in Simulation. 1986;28(6):453–466. 10.1016/0378-4754(86)90040-6

[19] Abdullah AR. The four point explicit decoupled group (EDG) method: a fast Poisson solver. International Journal of Computer Mathematics. 1991;38:61–70. 10.1080/00207169108803958

[20] Othman M, Abdullah AR. An efficient four points modified explicit group poisson solver. International Journal of Computer Mathematics. 2000;76:203–217. 10.1080/00207160008805020

[21] Ali NHM, Ng KF. Modified explicit decoupled group method in the solution of 2-D elliptic PDES. In: Proceedings of the 12th WSEAS International Conference on Applied Mathematics. MATH’07; 2007. p. 162–167.

[22] Young DM. Iterative Solution of Large Linear Systems. Academic Press; 1971.

[23] Mohanty RK, Kumar R, Dahiya V. Cubic spline iterative method for Poisson’s equation in cylindrical polar coordinates. ISRN Mathematical Physics. 2012;2012 10.5402/2012/234516

